# Coping Strategy, Life Style and Health Status During Phase 3 of Indian National Lockdown for COVID-19 Pandemic—A Pan-India Survey

**DOI:** 10.3389/fpubh.2022.814328

**Published:** 2022-05-18

**Authors:** Raghuram Nagarathna, Manjunath N. K. Sharma, Judu Ilavarasu, Ravi Kulkarni, Akshay Anand, Vijaya Majumdar, Amit Singh, Jagat Ram, Manjari Rain, Hongasandra R. Nagendra

**Affiliations:** ^1^Swami Vivekananda Yoga Anusandhana Samsthana, Bengaluru, India; ^2^Neuroscience Research Lab, Department of Neurology, Postgraduate Institute of Medical Education and Research, Chandigarh, India; ^3^CCYRN – Collaborative Centre for Mind Body Intervention Through Yoga, Post Graduate Institute of Medical Education and Research, Chandigarh, India; ^4^Centre of Phenomenology and Cognitive Sciences, Panjab University, Chandigarh, India; ^5^Department of Ophthalmology, Postgraduate Institute of Medical Education and Research, Chandigarh, India

**Keywords:** COVID-19, public health, stress, coping strategy, lockdown

## Abstract

The implementation of timely COVID-19 pan-India lockdown posed challenges to the lifestyle. We looked at the impact of lifestyle on health status during the lockdown in India. A self-rated scale, COVID Health Assessment Scale (CHAS) was circulated to evaluate the physical health or endurance, mental health i.e. anxiety and stress, and coping ability of the individuals under lockdown. This is a pan-India cross-sectional survey study. CHAS was designed by 11 experts in 3 Delphi rounds (CVR = 0.85) and was circulated through various social media platforms, from 9th May to 31st May 2020, across India by snowball circulation method. CHAS forms of 23,760 respondents were downloaded from the Google forms. Logistic regression using R software was used to compare vulnerable (>60 years and with chronic diseases) with non-vulnerable groups. There were 23,317 viable respondents. Majority of respondents included males (58·8%). Graduates/Postgraduates (72·5%), employed (33·0%), businessmen (6·0%), and professionals (9·7%). The vulnerable group had significantly (OR 1.31, *p* < 0.001) higher representation of overweight individuals as compared to non-vulnerable group. Regular use of tobacco (OR 1.62, *p* = 0.006) and other addictive substances (OR 1.80, *p* = 0.039) showed increased vulnerability. Respondents who consume junk food (OR 2.19, *p* < 0.001) and frequently snack (OR 1.16, *p* < 0.001) were more likely to be vulnerable. Respondents involved in fitness training (OR 0.57, *p* < 0.001) or did physical works other than exercise, yoga, walk or household activity (OR 0.88, *p* = 0.004) before lockdown were less likely to be vulnerable. Majority had a very good lifestyle, 94.4% never smoked or used tobacco, 92.1% were non-alcoholic, 97.5% never used addictive substances, 84.7% had good eating habits, 75.4% were vegetarians, 82.8% had “good” sleep, 71.7% did physical activities. Only 24.7% reported “poor” coping ability. Depression with somewhat low feeling were more likely to be vulnerable (OR 1.26, *p* < 0.001). A healthy lifestyle that includes healthy eating, proper sleep, physical activeness and non-addictive habits supports better coping ability with lesser psychological distress among Indian population during lockdown.

## Introduction

The past two decades have witnessed three highly pathogenic, novel zoonotic CoVs, first SARS-CoV-1 was recognized in 2002, followed by MERS-CoV in 2012 and now as a more virulent strain, the SARS-CoV-2 causing COVID-19 (1). Based on the estimated report of the instantaneous reproduction number (R_t_) on the severity in China, several countries implemented social distancing, hygiene etiquettes, contact tracing, wearing face masks, temperature checks, and avoided premature relaxation of the lockdown (2). The largest timely lockdown was enforced in India after its first case on 30th Jan 2020 (3).

The present COVID-19 pandemic affected global mental health, as evidenced initially by panic-buying, worldwide. Following any natural disaster, survivors are prone to develop post-traumatic stress disorder (PTSD). For instance, survivors of the August 2008 floods in India (Bihar) had shown higher scores for PTSD (4). Similarly, studies in China showed high level of depression with low health-related quality of life (HRQoL) and high scores on PTSD symptoms with no significant changes during COVID-19 (5, 6).

The first large-scale community-based cohort study on 387,109 adults in UK concluded that an unhealthy lifestyle (smoking, physical inactivity, obesity, and excessive alcohol intake) is a risk factor for hospital admission for COVID-19 (7). A study on Italian children observed increased screen time and sleep time, increased consumption of potato chips, red meat, and sugary drink and decrease in time spent in sports activities, which may have lasting impact on adiposity (8). The incidence, progression and death rate during this pandemic in India seems to be much lesser than other countries [COVID-19 Worldwide Dashboard | WHO Live World Statistics]. The reasons being India's relatively younger population, early biggest national lockdown (9), and a unique mutation in the spike surface glycoprotein [A930V (24351C > T)] in the Indian SARS-CoV-2 (10). A recent survey in Indian cohort showed that the level of psychological distress was lesser than the Chinese population on IES-R (11). There are unpublished observations that the traditional life style of the Indian families may also be a contributory factor. As there were no nationwide studies looking at the impact of life style on vulnerability during first wave and lockdown, we executed this pan-India on-line survey. The objective of the study was to investigate physical and mental health, lifestyle and to examine activities adapted by people to cope with COVID-19and lockdown.

## Materials and Methods

### CHAS Survey and Study Subjects

This was a nationwide survey on general Indian population during the 3rd phase of lockdown for COVID-19 pandemic that had respondents from all States/Union Territories except Ladakh and Lakshadweep. COVID Health Assessment Scale (CHAS), prepared in 10 languages by a committee of 11 experts through 3 Delphi rounds [Content Valid Ratio (CVR) was 0.85], had questions related to life style behavior (exercise, diet, additive substances, and sleep), physical health (BMI, chronic diseases, and endurance), mental health (fear, anxiety, depression, stress), and coping ability [refer to CHAS questionnaire from reference (12)]. Endurance under physical health signifies durability or ability to perform physical work for longer duration of time without feeling breathlessness. Further, coping ability is defined as conscious and unconscious efforts and strategies acquired by respondents such as reading, cooking and others to reduce emotional impact of challenging situation created by pandemic and lockdown.

Phone calls and special requests were sent to different sections of the society (~200 universities, corporate companies, healthcare institutions, government organizations, wellness centers, and their networks) to acquire data by snowball method. Participants filled the online forms, if they were willing to answer the subjected questions. Hence, there were no exclusion and inclusion criteria for participants.

The responses were collected from May 9, 2020 to May 31, 2020. Responses from non-Indians and aged <18 years were not considered for analysis. After quality control, the participants were divided into vulnerable and non-vulnerable groups based on presence of co-morbidities and age >60 years. Respondents were considered vulnerable when their age was above 60 years and/or they have any chronic disease as these two conditions increases the risk of getting infected with COVID-19 and risk of severe outcome. For zone wise analyses, the 34 states/UTs were divided into 3 zones based on the number of positive cases in the state as on 31st May 2020 (source: Ministry of health and Family Welfare, Government of India). The groups were red zone (>10,000 cases including Maharashtra, Tamil Nadu, Delhi, Gujarat), orange zone (5,000 to 10,000 cases including Rajasthan, Madhya Pradesh, Uttar Pradesh), and green zone (<5,000 cases including remaining states and UTs).

### Statistical Analysis

The CHAS data received from the Google drive in ten languages were combined into one dataset. R Statistical software, version 4.0.0, was used for data cleaning, extraction, and analyses. Incomplete and unreliable responses were excluded. Logistic regression was used to compare respondents under two categories viz. vulnerable and non-vulnerable. Arsenal package was used to test significance on cross tabulations on categorical variables. Reference for Odd's ratio (OR) calculation was set to sequential contrast for all ordinal variables and first row and first column for nominal variables.

## Results

Of the 23,760 respondents, participants from other countries (*n* = 401) and marked as other genders (*n* = 42) were excluded. Data was analyzed for 23,317 respondents. Logistic regression to compare 4,416 vulnerable participants with 18,901 non-vulnerable participants showed that graduates (OR 0.77, *p* < 0.001) were less likely to and postgraduates (OR 1.11, *p* = 0.027) were more likely to be associated with vulnerability than non-graduates (Table 1). Students were less likely to be associated with vulnerability than agriculturists (OR 0.26, *p* < 0.001). Businessmen (OR 1.87, *p* < 0.001), homemakers (OR 2.91, *p* < 0.001), professionals (OR 1.72, *p* < 0.001) and those in other occupations (OR 1.40, *p* = 0.005) were more likely to be vulnerable as compared to agriculturists (Table 1). Of note, only 3.2% were agriculturists and 48.7% were actively working professionals (6.0% business, 9.7% professionals, 33.0% employees) among the total respondents.

**Table 1:** Comparison of demographic details of vulnerable with non-vulnerable groups.

**Variables**	**Variable**	**Non- vulnerable** **(*n* = 18,901)**	**Vulnerable (*n* = 4,416)**	**Total** **(*n* = 23,317)**	**Odd's Ratio**	**CI: 2.5%**	**CI: 97.5%**	***p*-Value**
Gender	Female	7,727 (40.9%)	1,883 (42.6%)	9,610 (41.2%)	Ref			
	Male	11,174 (59.1%)	2,533 (57.4%)	13,707 (58.8%)	1.10	1.00	1.21	0.052
States zones*	Red	6,925 (36.7%)	1,731 (39.2%)	8,656 (37.2%)	Ref			
	Orange	4,215 (22.3%)	825 (18.7%)	5,040 (21.6%)	0.99	0.89	1.10	0.839
	Green	7,737 (41.0%)	1,857 (42.1%)	9,594 (41.2%)	1.02	0.93	1.13	0.663
Occupation	Agriculture	647 (3·4%)	101 (2·3%)	748 (3.2%)	Ref			
	Business	1.070 (5.7%)	321 (7·3%)	1,391 (6.0%)	1.87	1.47	2.41	<0.001
	Employed	6,648 (35.2%)	1,036 (23.5%)	7,684 (33.0%)	1.18	0.95	1.48	0.152
	Homemaker	1,916 (10.1%)	891 (20.2%)	2,807 (12.0%)	2.91	2.29	3.73	<0.001
	Student	4,377 (23.2%)	188 (4.3%)	4,565 (19.6%)	0.26	0.20	0.34	<0.001
	Professional	1,831 (9·7%)	436 (9·9%)	2,267 (9·7%)	1.72	1.36	2.20	<0.001
	Other	2,142 (11.3%)	449 (10.2%)	2,591 (11.1%)	1.40	1.11	1.79	0.005
Education	Less than Graduation	5,127 (27.1%)	1,296 (29.3%)	6,423 (27.5%)	Ref			
	Graduate	7,569 (40.0%)	1,640 (37.1%)	9,209 (39.5%)	0.77	0.70	0.84	<0.001
	Post-graduate	6,205 (32.8%)	1,480 (33.5%)	7,685 (33.0%)	1.11	1.01	1.21	0.027
During lockdown staying with	Family	15.781 (83.5%)	3,942 (89.3%)	19,723 (84.6%)	Ref			
	Friends	278 (1.5%)	32 (0.7%)	310 (1.3%)	0.62	0.41	0.90	0.017
	Colleagues	903 (4.8%)	79 (1.8%)	982 (4.2%)	0.41	0.31	0.52	<0.001
	Alone	1,064 (5.6%)	254 (5.8%)	1,318 (5.7%)	1.01	0.86	1.18	0.947
	Away from home	875 (4.6%)	109 (2.5%)	984 (4.2%)	0.41	0.52	0.81	<0.001
During lockdown are you	Working from home	7,474 (39.5%)	1,479 (33.5%)	8,953 (38.4%)	Ref			
	Working from office	4,185 (22.1%)	604 (13.7%)	4,789 (20.5%)	0.79	0.70	0.88	<0.001
	Not working	7,242 (38.3%)	2,333 (52.8%)	9,575 (41.1%)	1.22	1.12	1.33	<0.001
Are you experiencing any of the following?	No symptoms	16,866 (89.2%)	3,791 (85.8%)	20,657 (88.6%)	Ref			
	Cough	202 (1.1%)	66 (1.5%)	268 (1.1%)	2.08	1.52	2.81	<0.001
	Fever	27 (0.1%)	5 (0.1%)	32 (0.1%)	1.24	0.41	3.06	0.674
	Breathing Difficulty	23 (0.1%)	24 (0.5%)	47 (0.2%)	7.52	3.95	14.37	<0.001
	Other	1.783 (9.4%)	530 (12.0%)	2.313 (9.9%)	1.74	1.55	1.95	<0.001
Have you undertaken International travel since January 2020?	Yes	372 (2·0%)	110 (2·5%)	482 (2·1%)	Ref			
	No	18,529 (98.0%)	4,306 (97.5%)	22,835 (97.9%)	1.01	0.79	1.30	0.969

* *States in red zone (>10,000 positive cases in the state)—Maharashtra, Tamil Nadu, Delhi, Gujarat; orange zone (5,000 to 10,000 cases)—Rajasthan, Madhya Pradesh, Uttar Pradesh; and green zone (<5,000 cases)—All other states and Union Territories. Odds ratio was calculated using sequential contrasts for ordinal variables and odds ratio calculated with first row and first column as reference for nominal variables. Ref indicates reference group*.

During the lockdown, those who were not working were more vulnerable than who worked from home (OR 1.22, *p* < 0.001). Those who had stayed away from home (OR 0.41, *p* < 0.001) or with friends (OR 0.62, *p* = 0.017) or colleagues (OR 0.41, *p* < 0.001) were less likely to be in the vulnerable group as they were younger and did not have illnesses. Individuals experiencing symptoms like cough (OR 2.08, *p* < 0.001), breathing difficulty (OR 7.52, *p* < 0.001) and others (OR 1.74, *p* < 0.001), except fever, were more likely to be vulnerable (Table 1).

### Life Style

Table 2 summarizes the life style variables. Good eating habits was reported by 84.7% and strict vegetarian diet was reported by 75.4% of the total respondents. Vulnerable group had better eating habit (0.89, *p* = 0.032) and more strict vegetarians (OR 0.49, *p* < 0.001). However, consumption of junk food (OR 2.19, *p* < 0.001) and frequent snacking (OR 1.16, *p* < 0.001) was positively associated with vulnerability.

**Table 2:** Lifestyle in vulnerable and non-vulnerable groups.

**Domain**		**Variable**	**Non- vulnerable** **(*n* = 18,901)**	**Vulnerable (*n* = 4,416)**	**Total**	Odd's Ratio**	**CI: 2.5%**	**CI: 97.5%**	***p*-value**
Addictions	Tobacco	Never	17,807 (94.2%)	4,198 (95.1%)	22,005 (94.4%)	Ref			
		Occasionally	837 (4.4%)	146 (3.3%)	983 (4.2%)	1.09	0.89	1.33	0·381
		Regularly	257 (1.4%)	72 (1.6%)	329 (1·4%)	1·62	1·14	2·28	0·006
	Alcohol	Never	17,299 (91.5%)	4,165 (94.3%)	21,464 (92.1%)	Ref			
		Occasionally	1,509 (8.0%)	233 (5.3%)	1,742 (7.5%)	1.00	0.84	1.17	0·959
		Regularly	93 (0.5%)	18 (0.4%)	111 (0.5%)	0.77	0.40	1.43	0·431
	Substance use	Never	18,407 (97.4%)	4,332 (98.1%)	22,739 (97.5%)	Ref			
		Occasionally	378 (2.0%)	55 (1.2%)	433 (1.9%)	0.77	0.57	1.02	0·081
		Regularly	116 (0.6%)	29 (0.7%)	145 (0.6%)	1.80	1.02	3.12	0·039
	Increased substance abuse during lockdown	Yes	221 (1.2%)	34 (0.8%)	255 (1.1%)	Ref			
		No	5,273 (27.9%)	990 (22.4%)	6,263 (26.9%)	1.10	0.76	1.65	0·618
		Not Applicable	13,407 (70.9%)	3,392 (76.8%)	16,799 (72.0%)	1.21	0.83	1.80	0·34
Diet	Eat discipline before	Yes	15,852 (83.9%)	3,898 (88.3%)	19,750 (84.7%)	Ref			
		No	3,049 (16.1%)	518 (11·7%)	3,567 (15.3%)	0·89	0·80	0·99	0·032
	Strict vegetarian/vegan	Yes	13,732 (72.7%)	3842 (87.0%)	17574 (75.4%)	Ref			
		No	5,169 (27.3%)	574 (13.0%)	5,743 (24.6%)	0.49	0.44	0.54	<0·001
	I like eating junk food	Yes	4,684 (24·8%)	454 (10.3%)	5,138 (22.0%)	Ref			
		No	14,217 (75.2%)	3,962 (89.7%)	18,179 (78.0%)	2.19	1.96	2.45	<0·001
	I tend to frequently snack	Yes	7,273 (38.5%)	1,279 (29.0%)	8,552 (36.7%)	Ref			
		No	11,628 (61.5%)	3,137 (71.0%)	14,765 (63.3%)	1.16	107	1.25	<0·001
Sleep	Quality of sleep before lockdown	Good	15,707 (83.1%)	3,599 (81.5%)	19,306 (82.8%)	Ref			
		Ok	2,724 (14.4%)	731 (16.6%)	3,455 (14.8%)	1.31	1.17	1.47	<0·001
		Bad	470 (2.5%)	86 (1.9%)	556 (2.4%)	0.82	0.63	1.07	0·157
	Quality of sleep during lockdown	Good	14,964 (79.2%)	3,472 (78.6%)	18,436 (79.1%)	Ref			
		Ok	3,093 (16.4%)	799 (18.1%)	3892 (16.7%)	1.22	1.09	1.37	<0·001
		Bad	844 (4.5%)	145 (3.3%)	989 (4.2%)	0.96	0.77	1.18	0·685
Activity	Physical activity before lock-down	Did yoga	8,493 (44.9%)	2,377 (53.8%)	10,870 (46.6%)	Ref			
		Went fitness	1,271 (6.7%)	107 (2.4%)	1,378 (5.9%)	0.57	0.45	0.71	<0·001
		Went walking	3,546 (18.8%)	925 (20.9%)	4,471 (19.2%)	1.28	1.13	1.43	<0·001
		Did household	2,071 (11.0%)	417 (9.4%)	2,488 (10.7%)	0.88	0.75	1.03	0·102
		Other	3,520 (18.6%)	590 (13.4%)	4,110 (17.6%)	0.81	0.71	0.93	0·004
	Physical activity during lock-down	Yoga	9,864 (52.2%)	2,721 (61.6%)	12,585 (54.0%)	Ref			
		Fitness	893 (4.7%)	74 (1.7%)	967 (4.1%)	0.56	0.43	0.73	<0·001
		Walking	2,377 (12.6%)	534 (12.1%)	2,911 (12.5%)	0.96	0.84	1.10	0·591
		Household work	3,070 (16.2%)	622 (14.1%)	3,692 (15.8%)	0.94	0.82	1.07	0·36
		Other	2,697 (14.3%)	465 (10.5%)	3,162 (13.6%)	0.93	0.80	1.08	0·326
	Duration of the Activity During Lock-Down	Never	1,239 (6.6%)	256 (5.8%)	1,495 (6.4%)	Ref			
		<30 min	4,783 (25.3%)	890 (20.2%)	5,673 (24.3%)	0.79	0.68	0.93	0·004
		30 min−1 h	7,381 (39.1%)	1,703 (38.6%)	9,084 (39.0%)	1.15	1.05	1.26	0·004**
		> 1 h	5,498 (29.1%)	1,567 (35.5%)	7,065 (30.3%)	1.12	1.04	1.22	0·004**

*Odds ratio was calculated using sequential contrasts for ordinal variables and odds ratio was calculated with first row and first column as reference for nominal variables. Ref indicates reference group*.

Substance users were minimal in this cohort as majority said they “never” used tobacco (94.4%), or alcohol (92.1%) or other substances (97.5%) before lockdown; only 1.1% “agreed” they had increased the use of alcohol and tobacco during lockdown. Regular consumers of tobacco (OR 1.62, *p* = 0.006) and other substance users (very few in this cohort; OR 1.80, *p* = 0.039) were more likely to be vulnerable.

Looking at the quality of sleep, 82.8% and 79.1% said they had “good” sleep quality before and during lockdown, respectively in total respondents. Those who had average sleep before (OR 1.31, *p* < 0.001) or during (OR 1.22, *p* < 0.001) lockdown were more likely to be vulnerable than those who had good sleep. Individuals having bad sleep quality had increased from 2.4% to 4.2% during lockdown among total respondents.

Logistic regression further showed that those who went for fitness training (OR 0.57, *p* < 0.001) or did works other than exercise, yoga, walk or household activity (OR 0.88, *p* = 0.004) before lockdown were less likely to be vulnerable. Walking before lockdown did not reduce the risk of vulnerability (OR 1.28, *p* < 0.001). Individual practicing fitness training during the lockdown were less likely to be vulnerable (OR 0.56, *p* < 0.001). Very few individuals never did any physical activity during lockdown. Those who were involved in physical activity for <30 min (OR 0.79, *p* = 0.004) or for 30 to 60 min (OR 1.15, *p* = 0.004) were more in non-vulnerable group. Individuals involved in physical activity for more than an hour were more in vulnerable group (OR 1.12, *p* = 0.004). It is to be noted that 54.0% did yoga during lockdown while 46.6% were already practicing yoga (before lockdown).

### Physical Health

Majority had “good/average” endurance as marked on a 3 point scale (good, average, and bad). Respondents with average (OR 1.72, *p* < 0.001) and bad (OR 1.55, *p* = 0.014) endurance were more likely to be vulnerable than respondents who had good endurance. BMI was high in vulnerable group at 25·49 ± 4·34 kg/m^2^ than non-vulnerable at 24·05 ± 4·31 kg/m^2^ group (*p* < 0.001). The BMI between 23 and 24.9 kg/m^2^ (OR 1.63, *p* < 0.001) and above 25 kg/m^2^ (OR 1.31, *p* < 0·001) were more likely to be vulnerable (Table 3).

**Table 3:** Comparison of health and coping between vulnerable and non-vulnerable groups.

**Domain /Variable**		**Non- vulnerable** **(*n* = 18,901)**	**Vulnerable (*n* = 4,416)**	**Total N (%)**	**OR**	**CI: 2.5%**	**CI: 97.5%**	***p*-value**
**Physical health**								
How do you rate your physical strength and endurance?	Good	16,973 (89.8%)	3,662 (82.9%)	20,635 (88.5%)	Ref			
	Average	1,824 (9.7%)	693 (15.7%)	2,517 (10.8%)	1.72	1.55	1.89	<0.001
	Bad	104 (0.6%)	61 (1.4%)	165 (0.7%)	1.55	1.09	2.18	0.014
BMI	(N) mean ± SD	(17,666) 24.05 ± 4.31	(4,139) 25.49 ± 4.34					<0.001
	<23	7,489 (42.4%)	1,140 (27.5%)	8,629 (39.6%)	Ref			
	23–24·9	3,703 (21.0%)	903 (21.8%)	4,606 (21.1%)	1.63	1.48	1.79	<0.001
	>25	6,474 (36.6%)	2,096 (50.6%)	8,570 (39.3%)	1.31	1.20	1.43	<0.001
**Mental health**								
Depression: Do you feel you are low in energy and downhearted during this lock-down period?	Not at all	15,653 (82.8%)	3,735 (84.6%)	19,388 (83.1%)	Ref			
	Somewhat	3,000 (15.9%)	629 (14.2%)	3,629 (15.6%)	1.26	1.13	1.40	<0.001
	Very much	248 (1.3%)	52 (1.2%)	300 (1·3%)	1.06	0.77	1.45	0.718
Anxiety: How anxious are you about the implications of COVID-19 in your life?	Not at all	10,399 (55.0%)	2,856 (64.7%)	13,255 (56.8%)	Ref			
	Some what	6,495 (34.4%)	1,256 (28.4%)	7,751 (33.2%)	0.83	0.76	0.91	<0.001
	Very much	2,007 (10.6%)	304 (6·9%)	2,311 (9.9%)	0.89	0.77	1.03	0.113
**Fear: How much do the following issues worry you during this lock-down period?**								
Fear of getting infected and the associated physical suffering	Not at all	11,726 (62.0%)	2,982 (67.5%)	14,708 (63.1%)	Ref			
	Somewhat	5,818 (30.8%)	1,239 (28.1%)	7,057 (30.3%)	1.29	1.17	1.42	<0.001
	Very much	1,357 (7.2%)	195 (4.4%)	1,552 (6.7%)	0.97	0.80	1.17	0.741
Fear of death	Not at all	15,303 (81.0%)	3,802 (86.1%)	19,105 (81.9%)	Ref			
	Somewhat	2,825 (14.9%)	505 (11.4%)	3,330 (14.3%)	1.02	0.91	1.15	0.743
	Very much	773 (4.1%)	109 (2.5%)	882 (3.8%)	1.01	0.79	1.28	0.925
Fear of a possible financial burden	Not at all	10,359 (54.8%)	2,939 (66.6%)	13,298 (57.0%)	Ref			
	Somewhat	6,299 (33.3%)	1,130 (25.6%)	7,429 (31.9%)	0.74	0.67	0.80	<0.001
	Very much	2,243 (11.9%)	347 (7.9%)	2,590 (11.1%)	1.01	0.87	1.16	0.94
Fear of unknown related to COVID 19	Not at all	11,566 (61.2%)	3,110 (70.4%)	14,676 (62.9%)	Ref			
	Somewhat	5,625 (29.8%)	1,078 (24.4%)	6,703 (28.7%)	0.96	0.87	1.06	0.456
	Very much	1,710 (9.0%)	228 (5.2%)	1,938 (8.3%)	0.90	0.75	1.07	0.231
Fear of spreading infection to near and dear ones	Not at all	9,564 (50.6%)	2744 (62.1%)	12,308 (52.8%)	Ref			
	Somewhat	6,353 (33.6%)	1260 (28.5%)	7,613 (32.6%)	0.76	0.70	0.83	<0.001
	Very much	2,984 (15.8%)	412 (9.3%)	3,396 (14.6%)	0.79	0.69	0.91	0.001
Stress: Do you always feel insecure; stressed and have mood swings	disagree	12,470 (66.0%)	3,251 (73·6%)	15,721 (67.4%)	Ref			
	Maybe	4,117 (21.8%)	790 (17.9%)	4,907 (21.0%)	0.84	0.77	0.92	<0.001
	Agree	2,314 (12.2%)	375 (8.5%)	2,689 (11.5%)	0.83	0.72	0.95	0.007
Coping ability	Poor	4,727 (25.0%)	1,036 (23.5%)	5,763 (24.7%)	Ref			
	Good	14,174 (75.0%)	3,380 (76.5%)	17,554 (75.3%)	1.00	0.92	1.08	0.965
**How do you prefer spending time (apart from your regular, work-related engagements) during this national lock-down period?**								
TV	Yes	10,018 (53.0%)	2,188 (49.5%)	12,206 (52.3%)	Ref			
	No	8,883 (47.0%)	2,228 (50.5%)	11,111 (47.7%)	0.92	0.86	0.99	0.022
Read/Write	Yes	16,199 (85.7%)	3,676 (83.2%)	19,875 (85.2%)	Ref			
	No	2,702 (14.3%)	740 (16.8%)	3,442 (14.8%)	1.27	1.15	1.39	<0.001
Cook	Yes	13.658 (72.3%)	2,995 (67.8%)	16,653 (71.4%)	Ref			
	No	5,243 (27.7%)	1,421 (32.2%)	6,664 (28.6%)	1.28	1.19	1.38	<0.001
Exercise	Yes	14,962 (79.2%)	3,329 (75.4%)	18,291 (78.4%)	Ref			
	No	3,939 (20.8%)	1,087 (24.6%)	5,026 (21.6%)	1.25	1.15	1.37	<0.001
Yoga-asana	Yes	12,671 (67·0%)	2,903 (65·7%)	15,574 (66·8%)	Ref			
	No	6,230 (33.0%)	1,513 (34.3%)	7,743 (33.2%)	1.18	1.08	1.28	<0.001
Meditation	Yes	14,481 (76.6%)	3,838 (86.9%)	18,319 (78.6%)	Ref			
	No	4,420 (23.4%)	578 (13.1%)	4,998 (21.4%)	0.42	0.38	0.47	<0.001
Faith practice	Yes	14,095 (74·6%)	3,374 (76·4%)	17,469 (74·9%)	Ref			
	No	4,806 (25.4%)	1,042 (23.6%)	5,848 (25.1%)	0.97	0.90	1.06	0.497
Internet	Yes	14,353 (75.9%)	2,929 (66.3%)	17,282 (74.1%)	Ref			
	No	4,548 (24.1%)	1,487 (33.7%)	6,035 (25.9%)	1.50	1.39	1.62	<0.001

*Odds ratio was calculated using sequential contrasts for ordinal variables and odds ratio was calculated with first row and first column as reference for nominal variables. Ref indicates reference group*.

### Mental Health

Majority did not feel depressed (low feeling) in vulnerable group. Those with depression with somewhat low feeling were more likely to be vulnerable (OR 1.26, *p* < 0.001) (Table 3). Anxiety about implication of COVID-19 on life did not associate with increased vulnerability. Interestingly, who were “somewhat anxious” were less likely to be vulnerable (OR 0.83, *p* < 0.001) (Table 3).

We enquired on five aspects of fear. Fear of getting infected with COVID-19 and associated physical suffering was associated with vulnerability (OR 1.29, *p* < 0.001). Concerns about financial implications (OR 0.74, *p* < 0.001) and fear of infecting near and dear ones (OR 0.76, *p* < 0.001 for somewhat; OR 0.79, *p* = 0.001 for very much) were not associated with vulnerability (Table 3).

About 21% of the respondents were not sure about stress and insecurity, which did not associated with vulnerability (OR 0.84, *p* < 0.001). Those who agreed that they were stressed and insecure were less likely to be in vulnerable category (OR 0.83, *p* = 0.007).

### Coping

Coping ability was good in 75.3% and poor in 24.7% of the total respondents but was non-significant between vulnerable and non-vulnerable respondents.

Those who did not spend time reading (OR 1.27, *p* < 0.001) or cooking activity (OR 1.28, *p* < 0.001) or spend time on exercise (OR 1.25, *p* < 0.001), or did not do *Yogasana* (OR 1.18, *p* < 0.001) were more likely to be vulnerable (Table 3). The respondents who spend less time on internet were more likely to be vulnerable (OR 1.50, *p* < 0.001).

## Discussion

This first largest pan-India online survey, during the third phase of nation-wide Indian lockdown, looked at the life style, physical health, mental health and the coping abilities using logistic regression.

There is a well-established association between old age and co-morbidities such as hypertension (30%), diabetes (19%), and coronary heart disease (8%) with risk of COVID-19 infection (13–15). UK risk factor estimates had shown a dose-dependent increase in risk of COVID-19 with 4-fold higher risk in individuals with most adverse life style (51% of severely infected cases) compared to those with optimal lifestyle (7). Although our survey did not target COVID-19 positive cases, we looked at the potential associations between life style correlates of respondents, vulnerable to COVID-19 infection, with the non-vulnerable.

We observed similar gender distribution among vulnerable and non-vulnerable. However, it is reported that males are more vulnerable (13). Graduates/post graduates and those who had employment or business were more likely to be vulnerable than unemployed persons or agriculturists, who are physically active, similar to observations in China (16). This observation indicates that individuals having stressful job in urban regions make them vulnerable. It is important to state here that individuals residing in urban regions are at risk of getting infected, where day to day physical contact with each other is high, such as at offices, institutes, colleges, airport, markets, due to fast-pace life style. Good lifestyle before lockdown seems to have contributed to adopting healthy activities (reading, writing, cooking, yoga, exercise, and household) with good psychological coping ability during lockdown. Higher education and good life style seen in this cohort may not reflect the lifestyle or the behavioral characteristics of the general Indian population. The limitation being that response to a call on social media may reflect the social responsibility of those with good life style by responding voluntarily to this unstructured survey.

### Eating Habits

A healthy diet, rich in fruits and vegetables and low in sugar and calorie-dense processed foods, is essential to health, which was observed in majority of our respondents with majority being strict vegetarians. Diet rich in saturated fat, refined carbohydrates, and sugars with low levels of fiber that promotes obesity and type-2 diabetes poses increased risk for severe COVID-19 pathology and mortality by inhibiting adaptive immune system (17).

### Sleep

In a study, during the present pandemic, the prevalence of clinical insomnia in France was 19%, close to prevalence reported in China (20·1%) and Italy (19·8%) but lower than in Greece (37·6%) (18–21). We observed much lower prevalence with only 2·4% and 4·2% respondents reporting “bad” insomnia before and during lockdown. This appears to be because of the milder form of the disease in India and good family support.

### Addictions

Studies have observed that past or current smokers (18%) with COVID-19 had double the risk of progression to severe disease compared with never-smokers (9%) (22, 23). A small percentage (1.1%) who agreed that they had increased the consumption of addictive substances during lockdown did show increased vulnerability.

### Physical Health

Further, obesity is known to be an important contributor for many non-communicable diseases and also for respiratory and other infections (17). Pietrobelli et al. reported that children during social isolation in Italy gained weight which may have long term implications (8). We observed that the vulnerable group had higher representation of overweight individuals as compared to non-vulnerable group. Higher BMI denotes disturbed metabolism and an overall inflammatory state that could further increase the likelihood of COVID-19 infection in the vulnerable individuals (24).

Physical activity maintaining regular exercise with good physical endurance counteracts the negative effects of the pandemic stress on immune competency (25). In the present study, 71.7% did physical activities and those who did not do exercises and reported poor physical endurance were more likely to be vulnerable. Yoga, practiced by 54.0% of our respondents during lockdown, is an unexpected observation of this study (Table 2). Duggal et al. observed that exercise augments host immune defenses by catecholamine-mediated preferential mobilization of lymphocytes primed to recognize and kill virus-infected cells (26). Exercise also enhances proliferation of virus-specific memory T-cells and promotes their mobilization to the site.

### Mental Health

#### Anxiety and Depression

A meta-analysis of 65 studies during severe infections of SARS had noted that apart from the immediate mental health effects, PTSD could emerge at a later stage (27). “Somewhat” depressive low feeling and anxiety was noted in 15.6% and 33.2%, respectively while 1.3% and 9.9% were “very much” depressed and anxious, respectively. Similar observations have been reported by studies during this pandemic using different psychological battery. The first mental health survey in India during the initial phase of pandemic showed 33.2% had significant (mild/moderate/severe) psychological impact (11). Qiu et al. reported 29% had mild to moderate and 5% had severe psychological distress in China (28). Wang et al. reported psychological impact in higher percentage (53.8%) of respondents with higher stress scores that remained high in the 4th week (5). Thus, anxiety (28.8% in Wang et al. vs. 9·9% in present study) and depression (16·5% in Wang et al. vs. 1·3% in present study) seemed to be higher in China. This may be attributed to the good lifestyle in our respondents with higher educational level. In another Indian study, Rehman et al. observed that people who do not have enough supplies to sustain the lockdown were most affected and the affluent were negatively correlated with psychological distress (29).

#### Fear and Stress

A smaller percentage of respondents in our study were stressed (11.5%) or expressed “very much” fear of death (3.8%) or getting infected (6.7%) or financial burden (11.1%) as compared to Chinese respondents with high level of education (75%) similar to our cohort (72.5%), who experienced higher levels of stress (52.1% felt horrified and apprehensive) (15). Milder form of the disease, stricter lockdown and higher family support in Indian community may explain this difference.

The growing stress highlights the importance of funding translational and alternative medicine research (30) over fundamental research *in vitro* (31), *in vivo* (32–36) and biomarker studies (37–41), which is often restricted to publications. Translating this knowledge into practice may accelerate the pace of discovery and practice of integrative medicine. Several online surveys were conducted in India during this period; however, these surveys reported data on a small sample size (42–44), specific cohort (43, 45, 46), selective parameters such as psychological or life style or coping strategy (41, 44, 45, 47–49) as compared to our study. Another uniqueness of this study is that we used Delphi protocol to develop CHAS questionnaire for the survey.

### Limitations

Although, this survey was aimed at general population, the responses were received by only those with high level of education which prevents us from drawing any conclusion related to Indian race in general.

CHAS was prepared to suit the research question as we did not find a scale that had all the components we planned to assess. As the scale was self-reported, social desirability factor influencing the answers may be a limitation.

## Conclusion

This is the first nationwide large scale health survey, covering 34 states of India during the 3rd phase of lockdown of COVID-19 pandemic that shows that those with a good lifestyle including good eating, sleeping, and non-addictive habits with good physical activities adopt good coping abilities during the challenging times of life, irrespective of gender. Increased weight, unhealthy food, addictions and history of international travel increase the risk of getting infected with COVID-19 in vulnerable individuals. This study provides evidence for the media, policy makers and general population to include good life style recommendations for prevention.

## Data Availability Statement

The raw data supporting the conclusions of this article will be made available by the authors, without undue reservation.

## Ethics Statement

The studies involving human participants were reviewed and approved by Swami Vivekananda Yoga Anusandhana Samsthana, Bengaluru, India. Written informed consent for participation was not required for this study in accordance with the national legislation and the institutional requirements.

## Author Contributions

RN: concept, design, definition of intellectual content, literature search, manuscript preparation, manuscript editing, manuscript review, and guarantor. MS: concept, design, definition of intellectual content, manuscript preparation, manuscript editing, and manuscript review. RK: concept, design, definition of intellectual content, data acquisition, data analysis, statistical analysis, manuscript preparation, manuscript editing, and manuscript review. JI: concept, design, definition of intellectual content, data acquisition, data analysis, statistical analysis, manuscript editing, and manuscript review. AA: concept. VM: design, definition of intellectual content, literature search, data acquisition, manuscript editing, and manuscript review. AS: concept, design, definition of intellectual content, literature search, data acquisition, manuscript editing, and manuscript review. JR and MR: manuscript editing and manuscript review. HN: concept, design, definition of intellectual content, data acquisition, manuscript editing, and manuscript review. All authors contributed to the article and approved the submitted version.

## Conflict of Interest

The authors declare that the research was conducted in the absence of any commercial or financial relationships that could be construed as a potential conflict of interest.

## Publisher's Note

All claims expressed in this article are solely those of the authors and do not necessarily represent those of their affiliated organizations, or those of the publisher, the editors and the reviewers. Any product that may be evaluated in this article, or claim that may be made by its manufacturer, is not guaranteed or endorsed by the publisher.
